# Molecular Epidemiology and Clinical Manifestations of Adenovirus Respiratory Infections in Taiwanese Children

**DOI:** 10.1097/MD.0000000000003577

**Published:** 2016-05-06

**Authors:** Ya-Fang Wang, Fan-Ching Shen, Shan-Li Wang, Pin-Hwa Kuo, Huey-Pin Tsai, Ching-Chuan Liu, Jen-Ren Wang, Chia-Yu Chi

**Affiliations:** From the National Institute of Infectious Diseases and Vaccinology (Y-FW, F-CS, S-LW, J-RW, C-YC), National Health Research Institutes, Miaoli; Department of Pathology (P-HK, H-PT, J-RW), National Cheng Kung University Hospital; Department of Pediatrics (C-CL, C-YC), National Cheng Kung University Hospital; and Departments of Medical Laboratory Science and Biotechnology (J-RW), National Cheng Kung University, Tainan, Taiwan, R.O.C.

## Abstract

Human adenoviruses (HAdVs) are important causes of respiratory infections in children. They usually cause mild upper respiratory symptoms, but they can also produce severe pneumonia and other complications. The aims of this retrospective study were to better define the molecular epidemiology of respiratory adenoviruses circulating in Taiwanese children during 2002 and 2013, detect reinfections and co-infections, and characterize the clinical features and laboratory findings according to the causative genotypes.

We collected a representative sample of 182 isolates of adenoviruses from 175 children during the 12-year study period. The most prevalent species was HAdV-B genotype 3 (HAdV-3) (92/182, 50.5%) followed by HAdV-C (HAdV-2) (38/182, 20.9%). A single outbreak of HAdV-E (6/182, 3.3%) was noted in 2007. The mean age of children with adenovirus infections was 3.7 ± 2.0 years, with a slight predominance of males (53.1%). Children with HAdV-B tended to be older, had more lower respiratory tract infections, gastrointestinal symptoms, and a higher rate of hospitalization than those with HAdV-C (*P* < 0.05). Adenovirus co-infections were noted in 25/175 (14.3%) of the children. The most frequent co-infections were with species B (HAdV-3) and C (HAdV-2) (14/25, 56.0%). Additional infections were noted in 23/175 (13.1%) of the children. Of these repeated infections, the initial isolates were always genotypes of HAdV-C. The second isolates were genotypes of HAdV-B or HAdV-E. The clinical features of the first HAdV-B infection and the reinfection of HAdV-B followed the HAdV-C were similar.

In conclusion, HAdV-B, C, and E were the only adenovirus species that were isolated from children who were sufficiently ill with respiratory infections to require a visit to the hospital. Human adenovirus B (HAdV-3) accounted for half of these species. HAdV-B was more likely than other species to produce severe disease. The high incidence of adenovirus co-infection and reinfections with different HAdV species supports the need for continued surveillance and has major implications for development of vaccines.

## INTRODUCTION

Human adenoviruses (HAdVs) are responsible for a variety of infections in children. These range from mild, self-limited, upper respiratory infections to life-threatening disease in previously healthy, and also immunocompromised infants and young children,^1^ and those with underlying diseases.^2^ HAdVs can be isolated throughout the year in Taiwan.^4^ They are responsible for about 5% to 10% of lower respiratory tract infections and are the second most common viruses in children hospitalized with respiratory infections in this country.^3^

Human adenoviruses are double-stranded DNA viruses, belonging to the genus *Mastadenovirus* of the Adenoviridae family. More than 57 adenovirus serotypes have been identified. They are divided into 7 species (A–G). Different serotypes differ in their tissue tropisms and sites of infection. The most common adenovirus species that cause respiratory tract infections in children are B (HAdV-B3 and B7) and C (HAdV-C1, C2, and C5).^4^ Serotypes B3, B7, and B21 are the most frequent strains responsible for epidemics of acute febrile respiratory disease. The illnesses range from influenza-like fever and discomfort to pneumonia and death.^1,5,6^

Diagnosis of HAdV infection by viral isolation in cell culture is time-consuming and labor-intensive. Most clinical laboratories limit identification to HAdV, but do not determine the serotype or species-specific genotypes. The recent introduction of PCR-based assays has enabled rapid, specific, and sensitive detection of HAdV. In a previous study, using PCR assay systems targeting the hexon and fiber genes of HAdV, we reported that species B (HAdV-3) was the predominant respiratory adenovirus circulating in Taiwan over the past decade. A high incidence of co-infections and repeated infections were also observed. Nearly all children with repeated adenovirus infections had first infections with species HAdV-C, followed by HAdV-B or HAdV-E.^7^ The current study was designed to characterize the clinical features of infections with different adenovirus serotypes.

## METHODS

### Patients and Specimens

The population consisted of pediatric inpatients and outpatients who presented with signs and symptoms of acute respiratory tract infections to the National Cheng Kung University Hospital, from January 2002 to December 2013 from whom adenoviruses were isolated. These children were considered to be sufficiently ill to warrant throat swabs or nasopharyngeal aspirations for viral isolation. One to 2 isolates per month were randomly selected for this investigation regardless of the severity of their illness. Demographic data, clinical presentations, and outcomes were retrospectively reviewed. Only those patients in whom an adenovirus was the sole pathogen were included in the analysis.

This study was approved by the institutional review board (IRB) of the National Cheng Kung University Hospital (No. A-BR-101–020). This was a retrospective study without intervention or the need to obtain additional clinical specimens.

### Virology Studies

Throat swabs or nasopharyngeal aspirations were placed in virus transport medium and submitted to the virus diagnostic laboratory as soon as they were obtained, year-round. Viral cultures of laboratory-confirmed adenoviruses were processed as described previously.^8^ The adenoviruses were subcultured in A549 cells when 85% cytopathic effect was observed. The cells were then harvested for DNA extraction.

Viral DNA was extracted according to a modified procedure as previously described.^9^ DNA sequencing of hexon and fiber genes of respiratory adenovirus was carried out as previously described.^7^ Briefly, the Loop1 region of the hexon gene was amplified with primer pair HXL1F (5′-CGTGTGCAGTTYGCCCG) and HXL1R (5′-ACAGCCTGATTCCACAT). The Loop2 region of the hexon gene was amplified with primer BL (5′-TTGACTTGCAGGACAGAAA) and BR (5′-CTTGTATGTGGAAAGGCAC). PCR mixtures consisted of 1U of DNA polymerase (KOD Plus Polymerase, Toyobo), 1 mM MgSO_4_, 0.2 mM dNTP, 300 pm of each primer, and 1 to 2 μL of template from the original purified DNA solution in a 50-μL reaction volume. DNA sequencing analysis of PCR products was performed using Sanger method.

### Statistics

All analyses were performed with the statistical package from Social Sciences version 18.0 (SPSS Inc, Chicago, IL). The clinical characteristics of children with adenovirus respiratory infections were compared according to adenovirus species (Table 1) and for differences between outpatients and inpatients (Table 2). Continuous variables were compared by the *t* test or analysis of variance. Categorical data comparisons were performed by the chi-square test or Fisher exact test. *P* value less than 0.05 was considered to be statistically significant, and all tests were 2-tailed.

**TABLE 1:**
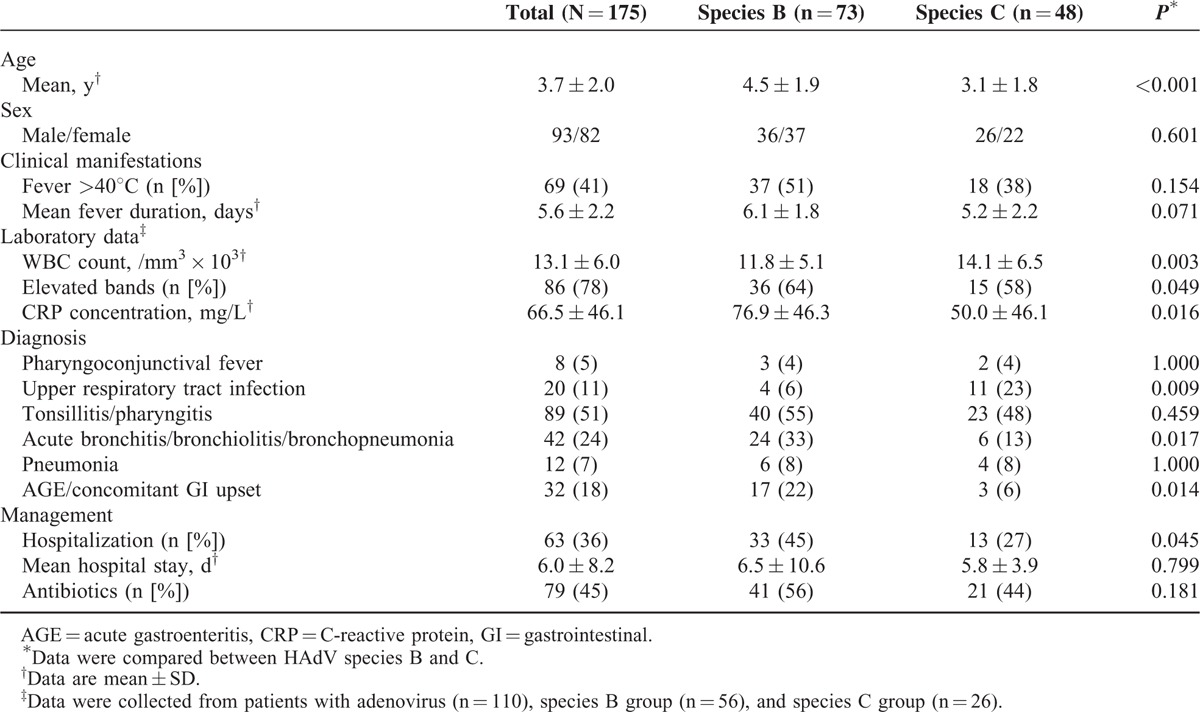
Clinical Characteristics of Children With Adenovirus Respiratory Infections According to Adenovirus Species

**TABLE 2:**
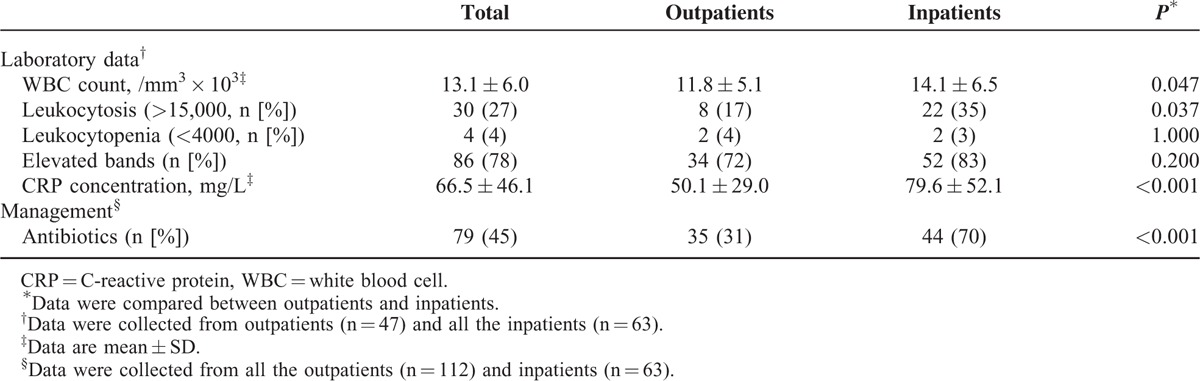
Laboratory Findings and Management of Children With Adenovirus Respiratory Infections

## RESULTS

### Virology Study

**FIGURE 1:**
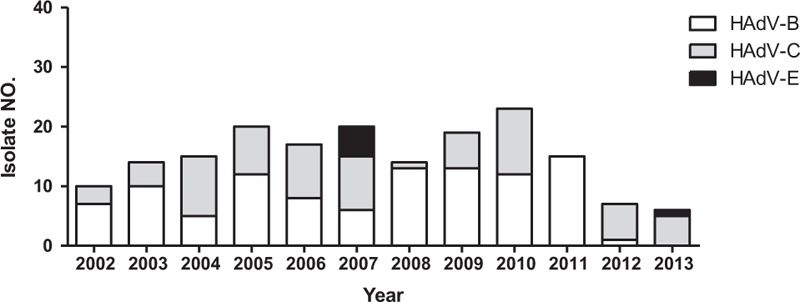
Distribution of respiratory adenoviral isolates in children with respiratory tract infections from 2002 to 2013 at a hospital in southern Taiwan.

Respiratory adenoviruses were identified in 2022 clinical specimens during the 12-year study. An average of 22 strains was isolated each month. One to 2 isolates for each month were randomly selected for this investigation. Of these, 182 could be propagated and underwent genotypic analysis. Eight genotypes were identified. These included species B (HAdV-3, 7, and 11), species C (HAdV-1, 2, 5, and 6), and species E (HAdV-4). Species B, mainly serotype 3, was the most common type, accounting for about half of the isolates (92, 50.6%), followed by species C (HAdV-2) (43, 23.6%). The yearly distribution of adenovirus serotypes during 2002 to 2013 is shown in Figure 1. Species E (HAdV-4) appeared and peaked in 2007 only.

### Demographic and Clinical Characteristics of Children with Adenovirus Infections

The demographic and clinical characteristics of the 175 children with adenovirus infections according the 2 most prevalent species (HAdV-B and HAdV-C) are shown in Table 1. The mean age was 3.7 years. Most (89/%) were under 6 years of age. About a third (39%) was less than 3 years old. There was no significant difference in the proportion of males and females. Almost all the children with adenovirus presented with fever (97%). Complete blood counts were performed for 110 of the children. The mean white blood cell (WBC) count was 13.1 ± 6.0 × 10^3^/mm^3^. Leukocytosis (WBC >15.0 × 10^3^/mm^3^) was noted in 30 of 110 (27%) with band forms (>10% of total WBC) in 78% of the patients. The mean C-reactive protein (CRP) concentration was 66.5 mg/L. Antibiotics were prescribed in 45% of the children.

Patients infected with HAdV-B were significantly older than those infected with HAdV-C (*P* < 0.001). Those infected with HAdV-B had lower leukocyte counts (*P* = 0.003), but higher CRP levels (*P* = 0.016) and hospitalization rates (*P* < 0.05). Both HAdV-B and HAdV-C could cause pneumonia, but HAdV-B was more frequently associated with acute bronchitis, bronchiolitis, or bronchopneumonia accompanied by gastrointestinal symptoms (*P* < 0.05).

### Laboratory Findings and Management According to Site of Care

About a third of the patients (36%) were hospitalized. Complete blood counts were done in all of the hospitalized patients, and 47 of the 112 outpatients (Table 2). The frequency of leukocytosis and elevated CRPs was significantly greater among the inpatients than among the outpatients (*P* < 0.05). Inpatients also were more likely than outpatients to be treated with antibiotics (70% vs 31%, respectively; *P* < 0.001). None of the children received antiviral therapy or intravenous immunoglobulins.

### Reinfections

A total of 23 (13.1%) children had an additional adenovirus infection during the 12-year study period. We were able to propagate and genotype isolates from 11 of these children. The time intervals between these infections ranged from 6 months to 5 years. All were first infected with HAdV-C (HAdV-1, HAdV-2, or HAdV-5), followed by HAdV-B (HAdV-3) or HAdV-E (HAdV-4). There were no significant differences in clinical features, laboratory findings, hospitalizations, and antibiotic use between the HAdV-B infection only and the HAdV-B reinfection followed the HAdV-C (Table 3).

**TABLE 3:**
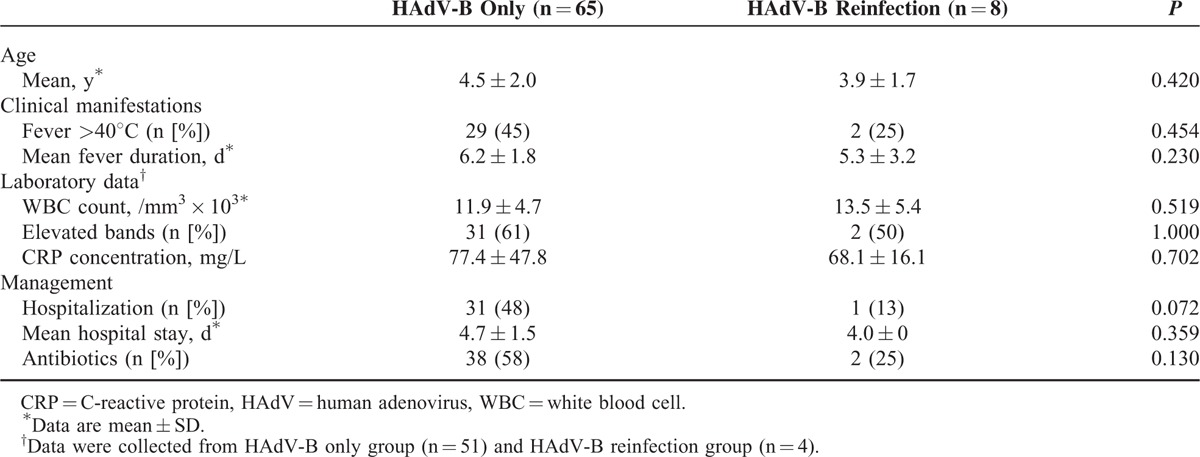
Clinical Manifestations of Children With HAdV-B Only and Reinfection Followed HAdV-C Respiratory Infections

### Co-infections

**FIGURE 2:**
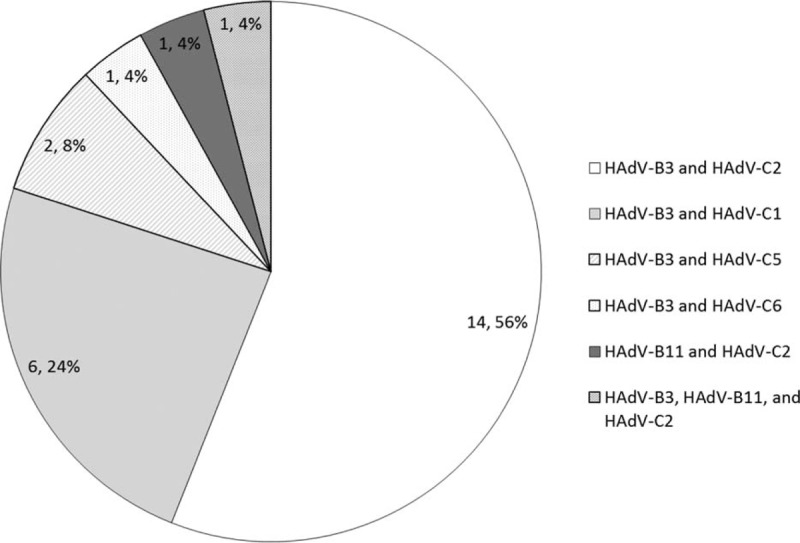
Number and frequency of adenoviral co-infections detected in children with respiratory tract infections.

A total of 25 children (14.3%) had HAdV co-infections. Most of those patients were co-infected with species B (HAdV-3) and species C (HAdV-2). The other duel combinations were species B (HAdV-3) and species C (HAdV-1); species B (HAdV-3) and species C (HAdV-5); and species B (HAdV-3) and species C (HAdV-6), species B (HAdV-11) and species C (HAdV-2). One patient was co-infected with 3 strains. These consisted of species B (HAdV-3), species B (HAdV-11), and species C (HAdV-2) (Figure 2).

## DISCUSSION

The aim of this 12-year retrospective study was to better understand the molecular epidemiology and clinical features of HAdVs in children in Southern Taiwan, and to determine whether there are important differences from other geographic regions. We found that only HAdV-B, C, and E were isolated from children with significant respiratory symptoms. HAdV species B and C co-circulate year-round and that children are often co-infected with genotypes of both species. Species B (HAdV-3) accounted for about half of the infections and was more likely than others to produce severe disease. In contrast, infections with species C were always found to precede those with species B even after long time intervals. Species E occurred much more episodically.

The mean age of our study population was 3.7 years. Most were younger than 5 years (77%), and only 23% were less than 2 years. This differs from previous reports that most HAdV infections occurred in children aged <2 years. The difference might be explained by the fact that most of the prior studies focused on hospitalized children with HAdV lower respiratory tract infections.^10–13^ The mean age of hospitalized patients with HAdV in our study was 3.5 years. The age differences among various studies might be attributed to several factors. Most Taiwanese children enter kindergarten after they are 3 years of age and exposed to respiratory infections. In addition, children with HAdV species C infections tend to be younger^10,14^ than those with species HAdV-B3, which was the most frequent isolate in our study.

Our findings are consistent with other studies that HAdV-B causes more severe respiratory tract infections than other serotypes and is more likely to result in hospitalization.^15,16^ We found that children with HAdV-B were more likely than those with HAdV-C to have an acute inflammatory response (leukocytosis, band forms, and elevated CRP), and to be hospitalized for lower respiratory tract infections and gastrointestinal distress.

Human adenoviruses are well known to be associated with prolonged viral shedding.^17^ To help differentiate between persistence and reinfection, we restricted the analysis to clinical recurrences at intervals of greater than 6 months. The key findings were that HAdV-C always preceded infections with HAdV-B or HAdV-E, and prior infection with HAdV-C did not attenuate the clinical manifestations of subsequent infections with HAdV-B. Based on these observations, it seems that primary infection with HAdV-C protects against subsequent HAdV-C infections, but not against HAdV-B or HAdV-E. These findings suggest that HAdV-B might be protective against HAdV-C, but this needs to be confirmed by further investigations. These observations may have important implications for vaccine development.

Prior investigators have demonstrated adenoviral co-infections using PCR-based molecular typing.^18–21^ In the current study, we found a relatively high frequency of adenoviral co-infections with HAdV-B and HAdV-C using generic and type-specific primers, followed by DNA sequencing of hexon and fiber genes. Co-infection provides the opportunity for genomic recombination. Genomic instability and evolutionary pressure pose potential problems for the long-term efficacy of HAdV vaccines.^22^

Our findings in Taiwan are consistent with the global predominance of HAdV-B3 as the most common serotype implicated in notifiable adenoviral infections in children and adults.^1,23,24^ Importantly, HAdV-3 may cause fatal pneumonias in immune-competent patients.^3,25^ No antiviral drug has been approved to treat HAdV infection. Oral vaccines against HAdV types 4 and 7 have been used successfully to control adenoviral illness in recruit populations.^26^ New vaccines for HAdV types 4 and 7 have been developed, and phase 3 studies demonstrated their safety and efficacy.^27^ Although previous studies revealed that HAdV type 7 immunization could generate a significant increase in levels of neutralizing antibodies against HAdV-3,^28^ further studies on the cross-preventive interaction between vaccine strains and currently circulating strains and other serotypes are needed.

In conclusion, our study indicates species B, especially HAdV-3, was the most frequent respiratory adenovirus circulating in Taiwanese children during the past 12 years and was associated with lower respiratory tract infections. Most of these children were less than 5 years old. Co-infections and reinfections were relatively common. These results support the need for continued surveillance and development of vaccines with broad protective immunity.
